# A Mimetic Assay of Neutrophil Extracellular Trap Degradation Using YOYO-1-Stained DNA-Histone Surface Webs

**DOI:** 10.3390/cells14080615

**Published:** 2025-04-19

**Authors:** Katherine H. Nguyen, Midori L. Wasielewski, Srilakshmi Yalavarthi, Xianggui Qu, Jason S. Knight, Shuichi Takayama

**Affiliations:** 1 Wallace H. Coulter Department of Biomedical Engineering, Georgia Institute of Technology and Emory University, Atlanta, GA 30332, USA; katherine.nguyen@gatech.edu (K.H.N.); midori.wasielewski@gmail.com (M.L.W.); 2 The Parker H. Petit Institute for Bioengineering and Bioscience, Georgia Institute of Technology, Atlanta, GA 30332, USA; 3 Division of Rheumatology, Department of Internal Medicine, University of Michigan, Ann Arbor, MI 48109, USA; syalavar@med.umich.edu; 4 Department of Mathematics and Statistics, Oakland University, Rochester, MI 48309, USA; qu@oakland.edu

**Keywords:** neutrophil extracellular traps, neutrophil extracellular trap degradation, assay development

## Abstract

Neutrophil extracellular traps (NETs) are not only promising biomarkers of disease, but also potential therapeutic targets. Overproduction or the improper clearance of NETs has been linked to disease severity. In vitro NET degradation assays can reveal mechanisms and degradation efficiency differences in diseased serum samples. There is a need for more convenient assays to increase the speed of NET degradation studies. This paper describes a simplified, lower variability mimetic assay with DNA–histone structures, referred to as surface webs, that performs functionally similarly to traditional NET degradation assays with increased scalability, ease of use, shorter preparation time, and lowered costs. The surface webs are created and dehydrated in a 96-well microplate that is shelf-stable, transportable, and viable for 30 days of storage at room temperature. The surface webs, compared to NETs, have similar shapes and distribution but lower intraplate variability while degrading with healthy serum and DNase I within the same timeframe. The assay can identify patient serum with reduced degradation capabilities. This assay opens new opportunities for NET-targeted drug discovery and studies on the role of NETs as modulators of disease.

## 1. Introduction

Neutrophil extracellular traps (NETs) are released by neutrophils through a process named NETosis in response to both foreign invasion and sterile inflammation-related stimuli [1,2,3]. NETs are primarily composed of DNA, histones, and proteins from neutrophil granules. Histones offer structural support for chromosomes and make up the majority of the proteins that are embedded in the decondensed DNA of NETs [4].

The discovered functions of NETs range greatly and are occasionally conflicting, as summarized by several review articles [3,5,6,7,8]. NETs have been shown to trap and kill bacteria [2,4,9], trap viral particles, and promote inflammation by recruiting and activating other cells [10,11,12]. Conversely, NETs may also decrease inflammation by sequestering and degrading cytokines and chemokines [13].

The contradictory inflammatory and anti-inflammatory properties of NETs highlight the complex roles and fragile balance of NETs in pathophysiology. Since neutrophils are abundant and highly mobile, the reach of NETs is extensive, having been found in both the bloodstream and tissues of many organs. NETs themselves are cytotoxic to the body’s own cells [14]. Thus, the dysregulation of NETs has the potential to play a role in a wide variety of pathologies.

Given their ability to cause cell or tissue damage, exaggerate inflammation, and trigger vaso-occlusion, tumor growth, and even autoimmune diseases, it is clear that the body must remove NETs once they have completed their initial job. NETs are typically degraded by a combination of deoxyribonucleases (DNase) DNase I and DNase IL3 found in serum [15]. Phagocytosis of NETs by macrophages has also been observed [16].

In fact, increased production or inefficient clearance of NETs is routinely connected to disease severity in several conditions, including inflammatory diseases, cardiovascular diseases, autoimmune diseases, lung diseases, and even cancer [3,17,18,19,20]. In a detailed review of NETs in disease, Papayannopoulos discusses how the presence of NETs can be harmful across disparate tissues and disorders [21]. Antiphospholipid syndrome (APS) is an example of a disease in which NETs appear to be pathogenic. APS is typically diagnosed in the setting of persistently detectable antiphospholipid autoantibodies (aPL) and a history of either thrombosis or pregnancy morbidity [22,23]. NETs are released when aPL accumulate on the neutrophil surface [24,25], are integral parts of aPL-associated thrombi [26], and are associated with more prevalent and severe thrombotic events [27]. Given NET involvement in APS, there is growing interest in investigating links between altered NET degradation and APS disease severity [28,29]

In vitro NET degradation assays are commonly used to compare how well serum from patients with various diseases can degrade NETs. While these degradation assays have furthered our understanding of disease pathophysiology, they are not without their weaknesses. Firstly, the assays are time intensive. All of the assays start with a blood draw, followed by neutrophil isolation, induction of NETosis, and, finally, the actual assay itself. Even producing the assays is low throughput. Established NET collection protocols show low yields of 700–900 ng of DNA from 1 × 10^6^ neutrophils and require several hours [30]. The time requirement is an issue, as the assays themselves are time sensitive. Neutrophils have a short half-life and require a fresh blood draw and collection for the NET generation. Once made, the NETs have a short shelf life; therefore, plates cannot be made in bulk. This requires additional planning for experiments and increases preparation time. Lastly, clinical samples are always highly variable. Clinical studies with NET degradation are hindered by the variability of NETs themselves. It has been shown that NETosis rates and predisposition to certain stimuli vary between donors [31]. Aside from donor-to-donor variability, NETs have varying amounts of types of miRNA, proteins, and DNA sources dependent on how NETosis is stimulated [32,33,34,35]. The varying functional components of NETs change how NETs interact with their environment and other cells, but can also affect NET removal, as DNase I only cleaves DNA that is accessible and free of protein [36]. Moreover, it is also possible to have some non-NET neutrophil remnants in the wells of the assay, for example, apoptotic material. Differences in neutrophil seeding and activation levels for NETosis will also contribute to well-to-well variability.

To tackle these issues, we build upon the current NET degradation assays to develop a mimetic degradation assay that utilizes a surface-bound DNA–histone structure (for brevity referred to as surface webs) that has a shorter preparation time, a lower resource cost, increased stability for storage, and lower variability. Using surface webs allows us to have greater control over the concentrations and compositions of the NET-like DNA–histone structures in each well while lowering the cost and experiment preparation time. We are able to easily generate milligrams worth of surface webs with commercially available isolated DNA and histone. We also compare the most commonly used DNA stain SYTOX Green with YOYO-1 and find the latter to maintain DNA fluorescence more stably, allowing intra-well DNA readings both before and after degradation. We also find that the surface webs pre-stained with YOYO-1 are degraded by DNase I and healthy human serum within the same timeframe as cell-derived NETs.

## 2. Materials and Methods

### 2.1. Selection of Literature for the Survey of Common Assay Methods

The survey of previously used assay methods is not comprehensive. A search was performed on Google Scholar, an electronic database of published literature, using the phrases “neutrophil extracellular trap degradation” and “neutrophil extracellular trap degradation patient”, sorted by relevance. Papers chosen were limited to serum-mediated degradation. Initial searches were not limited by date, but additional searches filtered to papers published since 2022 were conducted to include more current assay methods.

### 2.2. Surface Web Plate Fabrication

The following protocol to generate surface webs was adapted from our group’s previously published protocol to generate DNA–histone structures known as microwebs [37]. The protocol was adjusted for surface attachment and ease of storage and shipping.

DNA from salmon testes (Sigma Aldrich, St. Louis, MO, USA) and histone type II-A from calf thymus (Sigma Aldrich) were solubilized in phosphate-buffered saline (PBS). DNA and histone were combined in a total volume of 200 μL, with final concentrations of both at 500 μg/mL for a 1:1 DNA:histone ratio. Concentrations were measured using the Nanodrop One (Thermo Fisher Scientific, Waltham, MA, USA). The 200 μL DNA–histone mixture was then sonicated at 20% amplitude for 20 s using a Q125 Sonicator (QSonica, Newton, CT, USA). Following sonication, the microwebs were diluted to the desired concentrations.

Untreated black-walled, clear-bottom 96-well special optics plates (Fisher Scientific) were coated with 0.001% poly-L-lysine (PLL) (Sigma Aldrich, St. Louis, MO, USA). The sonicated microwebs were diluted, added to the microplate, and centrifuged for 10 min at 4000 RPM (2276 RCF) in a microplate swinging-bucket rotor (M-20, Thermo Fisher Scientific, Waltham, MA, USA). The supernatant was then removed, and the plate was dehydrated in a vacuum desiccator for 24 h.

### 2.3. Surface Web Degradation Assay

Controls for the assay included healthy human serum for comparison, nuclease buffer as the negative control, and DNase I as the positive control. For our healthy serum condition, we used two different pooled serum samples to cover a wider range of healthy patients: normal human healthy pooled serum (Sigma Aldrich, St. Louis, MO, USA), referred to as healthy control serum #1, and human healthy male type AB pooled serum (Sigma Aldrich, St. Louis, MO, USA), referred to as healthy control serum #2. Human healthy male type AB pooled serum generally has fewer antibodies, as it does not contain anti-A or anti-B antibodies or pregnancy-related antibodies. The serum was diluted to 10%. DNase I from bovine pancreas (Sigma-Aldrich, St. Louis, MO, USA) concentrations were measured in Kunitz/mL, labeled as unit (U/mL) for experiments and discussion. The dyes used were SYTOX Green (Thermo Fisher Scientific, Waltham, MA, USA) and YOYO-1 (Thermo Fisher Scientific, Waltham, MA, USA). The dyes were diluted in PBS, while all treatment conditions were diluted in nuclease buffer (pH 7.5, 10 mM Tris–HCl, 10 mM MgCl_2_, 2 mM CaCl_2_, and 150 mM NaCl).

A total of 1 μM of YOYO-1 or SYTOX Green was added to the dehydrated surface web plates and incubated for 30 min at 37 °C, 5% CO_2_, and 95% humidity. The plate was then washed once with PBS. New PBS was added back into the wells for initial imaging and fluorescence measurements. The PBS supernatant was then removed and replaced with treatment conditions. The plate was then incubated for 4 h at 37 °C, 5% CO_2_, and 95% humidity. Following the incubation step, the plate was washed three times with PBS, and 100 μL of PBS was added for final imaging and fluorescence measurements. The entirety of the solution was removed in each aspiration portion of the wash steps. For the majority of the experiments, the surface web plate wash steps were performed using the BioTek 50 TS Microplate Washer (Agilent BioTek, Santa Clara, CA, USA), with 100 μL of PBS per wash step. The aspirator of the microplate washer was positioned at the edge of the well. For experiments with human samples, the 100 μL washes were performed manually using a multichannel pipette angled to the edge of the well.

Images were acquired using the EVOS FL Auto 2 (Invitrogen, Waltham, MA, USA) inverted multi-channel fluorescence and transmitted light microscope. Fluorescence measurements before and after treatment were collected using the Synergy H4 Hybrid Multi-Mode Microplate Reader (Agilent BioTek, Santa Clara, CA, USA). Prior to reading, the microplate reader was warmed to 37 °C and the plates were incubated for 5 min in the machine to reduce temperature effects on fluorescence. Readings were taken as a 3 × 3 area scan at the bottom of the well. SYTOX Green readings were acquired using excitation at 504 nm, emission at 523 nm, a bandwidth of 9 nm, and a gain of 90. YOYO-1 readings were acquired using excitation at 485 nm, emission at 515 nm, a bandwidth of 13.5 nm, and a gain of 90.

### 2.4. Human Neutrophil Isolation

Human neutrophils were isolated as described previously [29,38]. Briefly, blood was fractionated by Ficoll/Hypaque gradient (Cytiva, Marlborough, MA, USA) to separate peripheral blood mononuclear cells from neutrophils. Neutrophils were then further purified by dextran sedimentation of the red blood cell (RBC) layer, before lysing residual RBCs with 0.2% sodium chloride.

### 2.5. Immunofluorescence Microscopy

A total of 1 × 10^5^ healthy control neutrophils were seeded onto 0.001% poly-L-lysine-coated (Millipore Sigma, St. Louis, MO, USA) coverslips, as described previously [29,38]. Briefly, neutrophils were incubated in RPMI media supplemented with L-glutamine and 1% fetal bovine serum (FBS), and NET formation was induced with 40 nM phorbol 12-myristate 13-acetate (PMA, Millipore Sigma) for 2 h at 37 °C and 5% CO_2_. Following stimulation, the cells were fixed with 4% paraformaldehyde for 10 min at room temperature, followed by overnight blocking in 10% FBS in PBS (blocking buffer). For protein staining, the fixed cells were incubated with a polyclonal antibody to neutrophil elastase (Millipore Sigma, 481001) in blocking buffer for 1 h at 4 °C, followed by an FITC-conjugated secondary antibody (Southern Biotech, Birmingham, AL, USA, 4052-02) for 1 h at 4 °C. DNA was stained with Hoechst 33342 (Invitrogen). Coverslips were mounted with Prolong Gold Antifade (Thermo Fisher Scientific), and images were acquired with a Cytation 5 Cell Imaging Multi-Mode Reader (BioTek).

### 2.6. NETosis Assay

NETosis was measured using a cell-impermeant dye, SYTOX Green (Thermo Fisher), as described previously [38]. Briefly, purified neutrophils were resuspended in 1× PBS (Gibco, Thermo Fisher Scientific, Waltham, MA, USA). A total of 10^5^ neutrophils were seeded into each well of a 96-well, black-walled, clear-bottom tissue culture plate (Costar, Corning, Corning, NY, USA) and were allowed to adhere for 20 min at 37 °C and 5% CO_2_. PBS was gently removed, and NET formation was induced with 40 nM PMA diluted in RPMI culture medium supplemented with L-glutamine and 1% FBS. SYTOX Green (Thermo Fisher Scientific) or YOYO-1 (Thermo Fisher Scientific) was added simultaneously to a final concentration of 1 µM. All treatments were conducted in triplicate. Cells were allowed to undergo NETosis for 2 h at 37 °C and 5% CO_2_. The culture medium was gently removed, and the wells were washed once with 1× PBS. Fresh PBS was added to each well, and residual NETs were quantified by measuring fluorescence at excitation and emission wavelengths of 504  nm and 523  nm, respectively, using a Cytation 5 Cell Imaging Multi-Mode Reader (BioTek). Data were collected using the area scan setting of the plate reader.

### 2.7. Human Samples

Patients were recruited at the University of Michigan in accordance with an approved IRB (HUM00122519) from the Institutional Review Boards of the University of Michigan, originally approved on 12 January 2017, with a renewal approval date of 15 January 2025. All patients provided their written informed consent before participation. Blood was collected in serum separator tubes (BD 367986) and centrifuged at 1300× *g* for 10 min at room temperature (18–25 °C). The serum was aliquoted and stored at −80 °C until further use.

### 2.8. Surface Web Degradation Assay with Antiphospholipid Syndrome Patient Serum

Surface web plates were fabricated as described in Section 2.2, then shipped from the Takayama Laboratory (Atlanta, GA, USA) to the Antiphospholipid Syndrome Research Laboratory (Ann Arbor, MI, USA). Once received, the plates were stored at room temperature until the experiments were performed. Six healthy human donor serum samples were pooled and used as a control. The assay protocol followed the methods Section 2.3, using 1 μM YOYO-1. Fluorescence was measured using a Cytation 5 Cell Imaging Multi-Mode Reader (BioTek) with excitation at 485 nm, emission at 515 nm, and the extended gain setting.

### 2.9. Statistical Analysis

All statistical tests were performed using GraphPad Prism (v10), with an α of 0.05. Central values depict the mean, while error bars depict the standard deviation. Shapiro–Wilk testing was used to determine normality. The respective statistical tests are described in figure legends.

## 3. Results

### 3.1. A Non-Comprehensive Survey of Serum- and Plasma-Mediated NET Degradation Assays

In Table 1, we summarize 24 different research groups’ assay workflows, and in Table 2, we summarize how the NETs were stained. The majority of methods to assess NET degradation use fluorescence-based microplate readers with some instances of using microscopy. Fifteen articles measured NETs that were still attached, while six articles measured NETs that were released into the supernatant. Data reporting methods and how conditions were compared varied, with most research groups choosing to normalize their readouts. However, the normalization method differed, with some normalized to a negative control, others to a positive control, and some normalized to both. Degradation times, where the NETs were exposed to experimental conditions, ranged from 1 to 21 h, with the majority in the 1-to-6-h range. Both serum and plasma were used, with concentrations of 10% used most often. From this survey, there were 12 uses of an untreated or buffer-only negative control and 14 uses of a positive control, either micrococcal nuclease (MNase) or DNase I. PicoGreen and SYTOX Green were the two most used dyes. Of note, six articles reported staining NETs prior to the incubation step and compared fluorescence before and after incubation.

Overall, there are many variations in methods and readouts, along with inconsistent presence of negative and positive controls, often only comparing healthy and diseased serum. This review of the literature highlights that there is no current standardized method to compare the efficacy of NET clearance. A consolidated literature review table can be found in Table S1 in the Supplementary File: survey of NET degradation assay methods.

### 3.2. Surface Webs Can Be Used as a Simplified Mimetic of NET Degradation Assays

**Figure 1:**
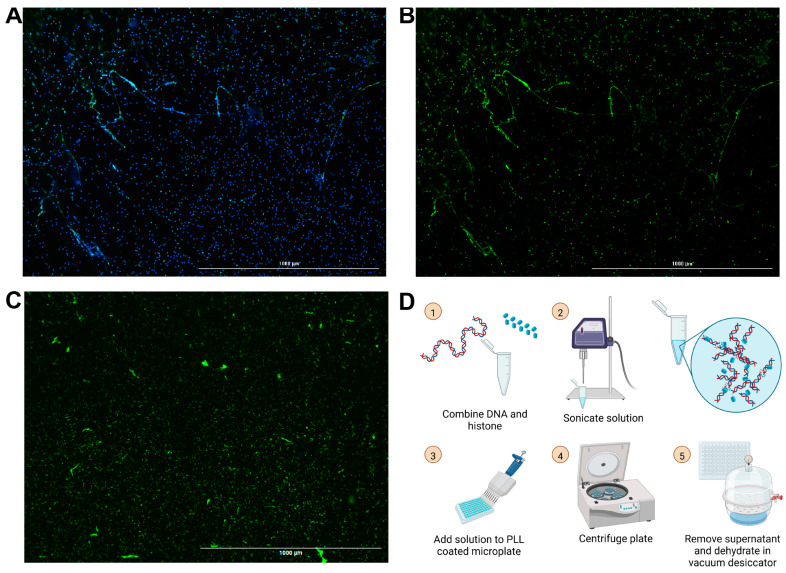
Images of NETs and surface webs. Neutrophils were stimulated with phorbol 12-myristate 13-acetate (PMA) to generate NETs. (**A**) Hoechst stain (blue) of DNA and neutrophil elastase stain (green) from PMA-stimulated neutrophils. (**B**) Neutrophil elastase stain of PMA-stimulated neutrophils. (**C**) YOYO-1 DNA stain of DNA in surface webs. (**A**–**C**) Scale bars = 1000 μm. (**D**) Schematic for surface web fabrication with steps numbered 1 through 5.

Building off of our group’s previously published chromatin microwebs that have been used as a NET mimetic, we adapted them to be suitable for degradation assays [37]. NET degradation assays require surface attachment of the DNA for ease in solution replacement and wash steps. The microwebs were diluted and centrifuged in poly-L-lysine (PLL)-coated 96-well microplates, so that the webs, now referred to as surface webs, were attached to the bottom of the wells (Figure 1D). The plate was then desiccated to increase its storage stability. This process generated structures (Figure 1C) shaped and distributed similarly to NETs (Figure 1A,B).

**Figure 2:**
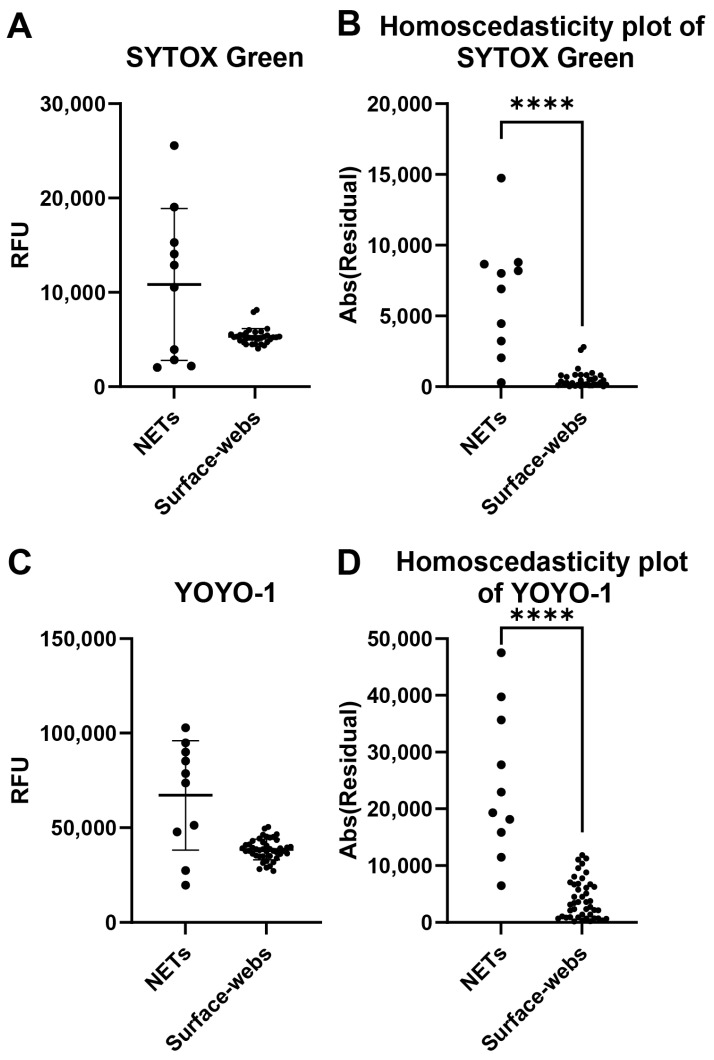
Well-to-well variability comparison of NETs and surface webs. Each data point shown is from a well within the same plate of NETs or surface webs. (**A**) Relative fluorescence units (RFU) of SYTOX-Green-stained NETs and surface web wells. Mean of NETs = 10,835 RFU, mean of surface webs = 5326 RFU. Standard deviation (SD) of NETs = 8048 RFU, SD of surface webs = 845.6 RFU. Coefficient of variation (CV) of NETs = 74.28%, CV of surface webs = 15.88%. (**B**) Homoscedasticity plot of data points from (**A**). Normality testing with Shapiro–Wilk revealed normally distributed data for NETs (W = 0.9115, *p* = 0.2915), but non-normally distributed data for surface webs (W = 0.8140, *p* < 0.0001). Levene’s median test showed significant differences between NET and surface web variances (**** *p* < 0.0001). (**C**) RFU of YOYO-1-stained NETs and surface web wells. Mean of NETs = 67,162 RFU, mean of surface webs = 38,512 RFU. SD of NETs = 28,925 RFU, SD of surface webs = 5411 RFU. CV of NETs = 43.07%, CV of surface webs = 14.05%. (**D**) Homoscedasticity plot of data points from (**B**). Normality testing with Shapiro–Wilk revealed normally distributed data for NETs (W = 0.9223, *p* = 0.3763) and surface webs (W = 0.9837, *p* = 0.7811). The F test showed significant differences between NET and surface web variances (F(9,43) = 28.57, **** *p* < 0.0001).

A common problem with NET degradation assay experiments is the well-to-well variability. Several factors may impact the variability, including neutrophil seeding, differences in neutrophil activation levels for NETosis, and remnant nuclear DNA contaminating the wells. We assessed individual well fluorescence readings of NETs and surface webs to compare the well-to-well variability of both (Figure 2). Wells were stained with either SYTOX Green or YOYO-1. The absolute values of the residuals were plotted to create homoscedasticity plots to compare variance (Figure 2B,D). With both dyes, NETs showed significantly higher variability in RFU between wells, thus indicating higher variability of DNA within a microplate compared to a microplate of surface webs.

### 3.3. The Surface Web Assay Degrades with Typical NET Degradation Controls as Expected Within the Same Timeframe as Neutrophil-Derived NETs

We designed a protocol for our surface web degradation assay that closely resembles common procedures for NET assays. We tested the surface web degradation assay with commonly used NET degradation controls and concentrations, with incubation times within the timeframe of other NET degradation assays (Table 1). To reduce the impact of varying initial DNA content within wells on the assay results, we compared the fluorescence of each well before and after the degradation incubation step. The desiccated surface web plate was stained with a nucleic acid stain and washed once with PBS. An initial fluorescent intensity area scan of each well in PBS was taken using a microplate reader. Then, the supernatant was replaced with the degradation conditions and incubated for 4 h at 37 °C. Following the incubation step, the plate was washed three times with PBS. A final fluorescence intensity area scan of each well was acquired again in PBS.

The remaining surface webs present in each well after degradation was calculated as the percent remaining with the following equation:

(1) $$\%\;Remaining=\frac{\left(Final\;well\;RFU-average\;background\;RFU\right)}{\left(Initial\;well\;RFU-average\;background\;RFU\right)}\times 100$$

DNase I from bovine pancreas (Sigma Aldrich) was used as a positive control, while a nuclease buffer was used as a negative control.

#### 3.3.1. Using YOYO-1 Rather than SYTOX Green Improves Sample Resolution and Differentiation

**Figure 3:**
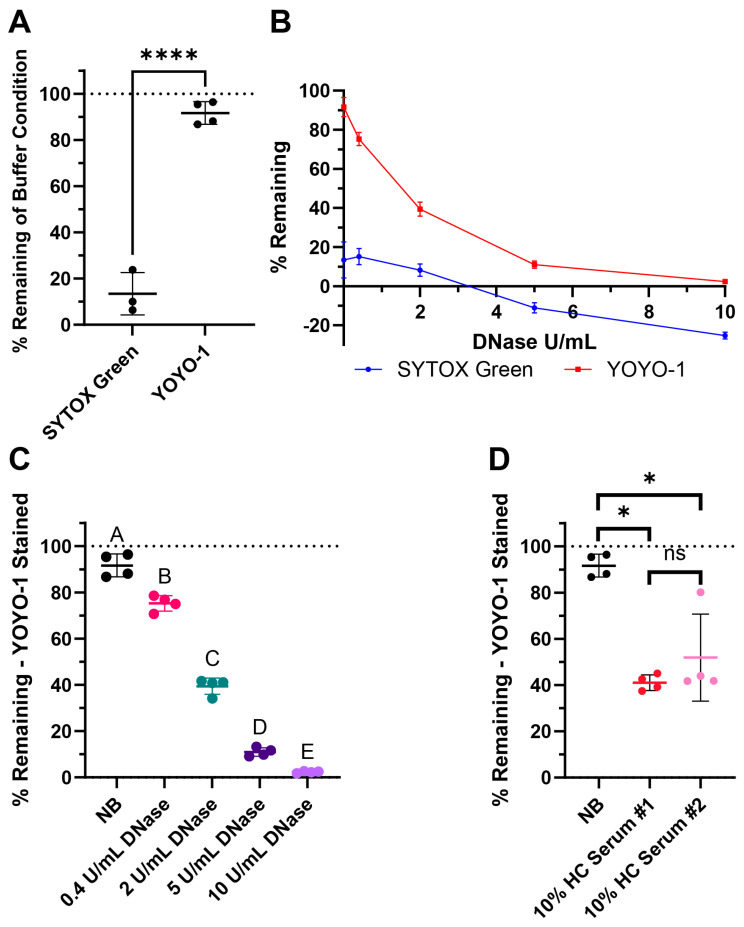
Surface web degradation assay tested with commonly used controls: DNase and pooled healthy control (HC) serum. (**A**) Comparison of SYTOX Green and YOYO-1 dye persistence with buffer-only conditions after 4 h, where the wells were stained before incubation. (**B**) DNase I curves from the treatment of 0.4, 2, 5, and 10 Kunitz/mL (U/mL) of DNase showing the possible dynamic range of the assay with respective dyes. (**C**) Assay results with the nuclease buffer (NB) and DNase I when stained with YOYO-1. (**D**) Assay results with NB and two pools of healthy control (HC) serum when stained with YOYO-1. (**A**–**D**) The line indicates the mean and the bars represent the standard deviation of the samples. (**A**) Normality testing with Shapiro–Wilk revealed normally distributed data (W = 0.9046, *p* = 0.3597). Two-sample two-tailed *t* test. **** *p* value < 0.0001. (**C**) Testing with Shapiro–Wilk revealed normally distributed data (W = 0.9517, *p* = 0.3935). Ordinary one-way ANOVA, followed by Tukey’s post-hoc test. Statistical significance is shown with compact letters, where groups with shared letters indicate *p* value > 0.05. Adjusted *p*-values are listed in Table S2. (**D**) Testing with Shapiro–Wilk revealed non-normally distributed data (W = 0.8001, *p* = 0.0095). Mann–Whitney test results for exact *p* values are as follows: between NB and HC Serum #1 * *p* = 0.0286, NB and HC Serum #2 * *p* = 0.0286, and HC Serum #1 and #2 *p* = 0.4857 labeled as not significant (ns). See Appendix A for an explanation of calculations and the existence of negative % remaining.

SYTOX Green is a commonly used dye in NET degradation assays (Table 2). YOYO-1 is an alternative nucleic acid stain with high affinity and low background signal. Here, we compared both dyes for our assay. When DNA was stained prior to the degradation step with SYTOX Green, the signal did not persist strongly in the nuclease buffer after 4 h of incubation (Figure 3A). When YOYO-1 was used instead, there was very little signal loss (Figure 3A). Furthermore, testing 0.4, 2, 5, and 10 Kunitz/mL (U/mL) of DNase I with both dyes created a smaller dynamic range when using SYTOX Green (Figure 3B). Notably, the % remaining of the buffer control and DNase conditions was noticeably lower compared to the same assay performed using YOYO-1. With the limited range between the positive and negative controls, there was less resolution between the degradation conditions.

#### 3.3.2. Degradation with DNase I and Two Pools of Healthy Human Serum

For our healthy control (HC) serum condition, we used two different pooled serum samples, normal human healthy pooled serum (Sigma Aldrich) as HC #1 and human healthy male type AB pooled serum (Sigma Aldrich) as HC #2.

The % remaining of buffer along with 0.4, 2, 5, and 10 U/mL DNase of YOYO-1 stained surface webs are shown in Figure 3C. % remaining of buffer and both 10% healthy pooled serum conditions are shown in Figure 3D with YOYO-1. There was a dose dependent response in DNase degradation as expected as higher concentrations of DNase degrade more DNA in the surface webs. The lowered % remaining in both pools of healthy human serum revealed healthy serum was able to degrade the surface webs within the 4 h, which is in the same timeframe as NET degradation. There was not a significant difference in the % remaining medians between the two healthy serum pools.

### 3.4. Surface Web Plates Are Viable After Storage at Room Temperature

We tested the storage stability of surface web plates while comparing storage at room temperature (RT) in the dark after 3, 7, 14, and 30 days. All plates were made on the same day using the same solution of webs and stored until the stated timepoint.

**Figure 4:**
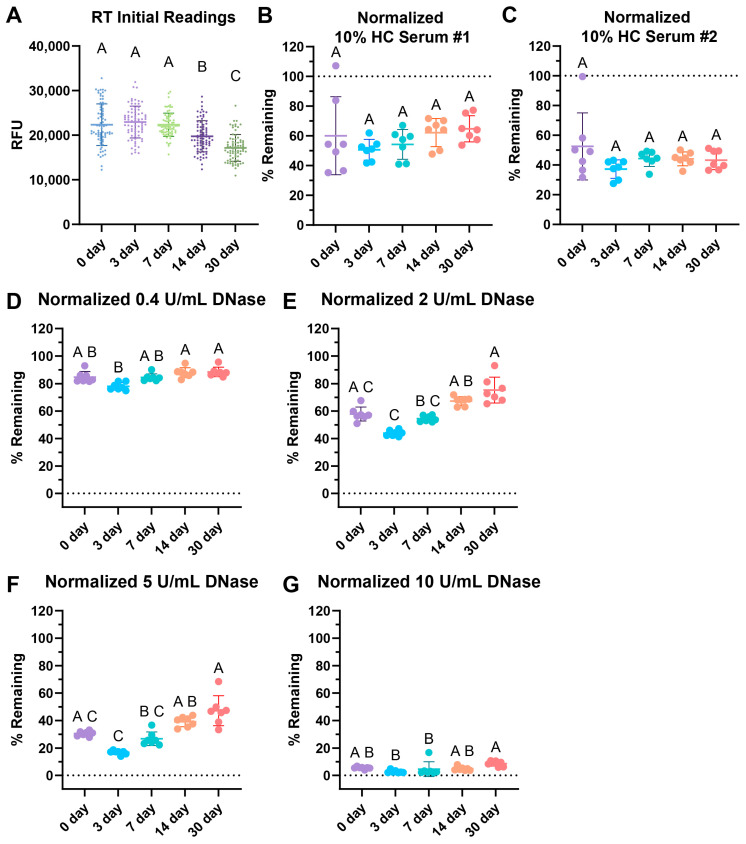
RT storage effects after 3, 7, 14, and 30 days. (**A**) Comparison of initial fluorescence readings over time, with 77 replicates per condition. Storage effects on (**B**) 10% healthy serum degradation with pool #1 healthy control (HC) serum, (**C**) 10% HC serum pool #2 degradation, (**D**) 0.4 Kuntiz/mL (U/mL) of DNase I. (**E**) 2 U/mL DNase I, (**F**) 5 U/mL DNase I, and (**G**) 10 U/mL DNase degradation. (**B**–**G**) Data normalized such that the negative nuclease buffer controls were 100% remaining. (**A**–**G**) Sample lines represent the mean and the bars the standard deviation. Kruskal–Wallis with post-hoc Dunn’s test. Statistical significance is shown using a compact letter display as a summary for multiple comparisons, where groups with shared letters indicate *p* values > 0.05. Adjusted *p*-values are listed in Tables S3–S9. Shapiro–Wilk testing reveals non-normality for (**A**) W = 0.9892, *p* = 0.0062; (**B**) W = 0.9254, *p* = 0.0204; (**C**) W = 0.7729, *p* < 0.0001; (**D**) W = 0.8897, *p* = 0.0021; (**E**) W = 0.9038, *p* = 0.0050; (**F**) W = 0.8294, *p* < 0.0001; and (**G**) W = 0.6901, *p* < 0.0001. See Appendix A for an explanation of calculations and the existence of over 100% remaining.

Interestingly, the initial readings of the wells dropped over time (Figure 4A). When tested with two different 10% healthy control (HC) serum pools, storage showed no significant differences over time with serum degradation up to 30 days of storage (Figure 4B,C). The healthy normal serum pool showed a slight, non-statistically significant increasing trend over time.

Degradation with 0.4, 2, 5, and 10 U/mL of DNase revealed some increasing difficulty in degradation with 2 and 5 U/mL of DNase over time with respect to RT storage (Figure 4E,F). However, the commonly used concentration of 10 U/mL DNase for positive controls, as seen in Table 1, was able to fully degrade the RT-stored plates for up to 30 days of storage (Figure 4G). This indicates that the positive control would still be viable had the assay been used, but the assay may lose sensitivity and degradation ability with lower concentrations of DNase.

### 3.5. Testing Antiphospholipid Syndrome Patient Serum Samples

**Figure 5:**
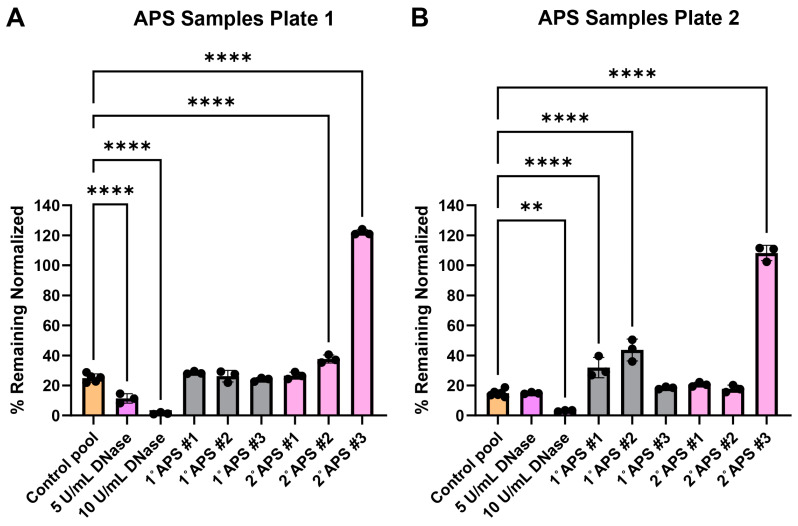
Surface web degradation with primary (1°) and secondary (2°) antiphospholipid syndrome (APS) patient serum. Data were normalized, such that negative nuclease buffer controls were 100% remaining. (**A**) Patient samples tested 15 days after fabrication. Normality testing with Shapiro–Wilk revealed normally distributed data (W = 0.9893, *p* value = 0.9246). Ordinary one-way ANOVA with post-hoc Dunnett’s test comparing conditions to the healthy serum control pool revealed significant differences among DNase conditions, 2° APS patient #1, and 2° APS patient #3. **** *p* value < 0.0001. (**B**) Patient samples tested 17 days after fabrication. Normality testing with Shapiro–Wilk revealed normally distributed data (W = 0.9470, *p* = 0.1401). Ordinary one-way ANOVA with post-hoc Dunnett’s test comparing conditions to the healthy serum control pool revealed significant differences among 10 U/mL DNase, 1° APS patient #1, 1° APS patient #2, and 2° APS patient #3. ** *p* value = 0.0018, **** *p* value < 0.0001. See Appendix A for an explanation of calculations and the existence of over 100% remaining.

The surface web plates were tested for their viability as a NET degradation assay mimetic with representative antiphospholipid serum (APS) patient serum samples after shipment. Past work using traditional NET degradation assays has demonstrated that some patients with APS have a defect in NET degradation [29]. Surface web plates were fabricated and shipped over 700 miles between Georgia Tech and the University of Michigan laboratories. The plates were used on day 15 (Figure 5A) and day 17 (Figure 5B) after fabrication. Serum samples from three primary (1°) and three secondary (2°, i.e., patients with concomitant lupus) APS patients were tested along with healthy control serum samples.

## 4. Discussion

As more research reveals the deleterious effects of the overabundance of NETs on disease severity, interest in the use of in vitro assays to measure NET degradation is growing. This study preliminarily validates an engineering solution to provide a more standardized, consistent, scalable, lower-cost, lower-preparation-time, and shelf-stable alternative to NET degradation assays that is functionally similar but not requiring neutrophils. We created DNA–histone structures, which we refer to as surface webs, that have a similar shape and distribution to NETs within 96-well plates. We found that the surface webs have lower well-to-well variability within a plate than NETs. Like NETs, the surface webs are degradable with healthy serum and DNase I. The surface web assay uses an incubation period of 4 h, which is within the same timeframe as that of other NET degradation assays, typically ranging from 1 to 21 h (Table 1). We also demonstrate that the surface webs are viable for assays for up to 30 days after plate dehydration. The plates could also be shipped to another laboratory for patient serum testing to identify patients who have reduced degradation abilities.

Multiple plates of surface webs can be quickly generated with commercially available isolated DNA and histone without the need for donor selection, blood draws, or neutrophil isolation steps. This not only lowers preparation time and cost, but also reduces neutrophil donor variability [31].

Our findings could also lower deviation within treatment groups for degradation assays by improving well uniformity and by showing that YOYO-1 dye is a more stable staining dye than SYTOX Green when pre-staining DNA attached to wells. We found that the absolute value of residuals of each well was statistically significantly lower with surface webs than NETs (Figure 2). The lower well-to-well variability of surface webs compared to NETs reduces the standard deviation of final assay results by having each replicate start with more even amounts of DNA per well. Additionally, we designed the surface web protocol such that the DNA is pre-stained prior to incubation with degradation conditions. Pre-staining allows us to take initial readings of each well as a reference when measuring degradation. Six of the articles surveyed, and reported in Table 2, also use this method. The other articles compare the final fluorescence of their treatment conditions with the final fluorescence of their control conditions. However, as shown and discussed previously, NETs have high variability in their starting amounts of DNA, due to the compounding differences from initial neutrophil seeding densities, activation levels for NETosis, and possible remnants of other neutrophil material affecting fluorescence. Comparing interwell rather than intrawell values may confound results due to the varying amounts of initial NET material for each experimental condition. Of the six papers that pre-stain, five of them use SYTOX Green (Table 2). In this study, we show that SYTOX Green signal does not persist as strongly as YOYO-1 after 4 h of incubation with a nuclease buffer (Figure 3). While still usable, the lower signal persistence leads to lower resolution and sensitivity between conditions. Both dyes are nucleic-acid-intercalating dyes and have properties that are well suited to NET degradation assays [59,60]. SYTOX Green and YOYO-1 are cell-membrane-impermeable dyes that are essentially nonfluorescent when not bound to nucleic acids. When bound, both dyes experience an approximately 1000-fold signal increase. YOYO-1’s better signal persistence when pre-staining may be due to its dimeric structure, very strong binding affinity, and high sensitivity [59,60,61].

We demonstrate how the surface web assay is viable after 30 days of storage at room temperature (Figure 4). The ability to store the plate greatly increases its ease of use, as multiple plates could be prepared beforehand and be immediately ready for use after the patient samples are drawn. This reduces the amount of coordination necessary to measure the degradation ability of diseased serum samples. Others have shown that they are able to store NETs for degradation assays overnight by keeping the NETs in PBS at 4 °C [15,58]. However, this is limited to overnight storage and does not allow for easy transport. The surface webs are dehydrated, making transportation and even shipping the plates possible.

Furthermore, the surface web plates could be shipped and stored to test patient serum degradation abilities. We tested the surface web assay with serum from three primary and three secondary APS patients. APS can be categorized as either primary or secondary, where primary APS patients do not present with any other immune disorders and secondary APS patients are associated with another immune disorder. The secondary patients in this study were associated with systemic lupus erythematosus (SLE). Previous research has found that a subset, but not all, of APS patients have reduced ability to degrade NETs [28]. The decreased ability of some patients to degrade NETs may be due to antibodies protecting NETs from DNases. Anti-NET antibodies found in APS patients correlated with impaired NET degradation ability and worse clinical manifestations [29]. Here, we also found that some APS patients were able to consistently degrade the DNA in surface webs while others could not. One out of three primary APS and one out of three secondary APS patients were able to consistently degrade as effectively as the healthy serum controls in both plates tested. The secondary APS patient #3 showed a severe inability to degrade the DNA in the surface webs, with the amount of surface webs remaining similar to that in the negative buffer control. The primary APS patients #1 and #2, along with the secondary APS patient #2, showed significant differences from the healthy serum control only in one of the two plates tested. This may indicate that these patients degrade the surface webs only moderately worse than healthy persons.

It may have been expected that the secondary APS patients who had SLE would demonstrate the most significant reduction in NET clearance abilities, as SLE patients have been shown to have impaired NET clearance in their own right [40,41,42,46]. Yet, in our findings, both primary and secondary categories had an equal number of patient sera able to degrade the surface webs, as well as healthy serum. It should be noted that our sample size was small for this pilot study, and more work will be needed to determine how primary and secondary APS patients compare in both surface web and traditional NET degradation assays. In SLE, impaired degradation correlates with disease activity and flares, where patients in remission are typically able to degrade NETs [41,42]. In fact, Leffler et al. also found that there was no difference in NET degradation ability between primary and secondary APS patients [28]. Thus, it is not unusual that some of the patient samples tested in this work were able to degrade NETs regardless of APS disease categorization. Taken together, this pilot study supports the further development of this and similar platforms for potential clinical use.

The surface webs are not the first engineered structure our group has designed for NET degradation assays. We have previously shown how DNA–histone mesostructures (DHMs) can be used in an image-based, wash-free degradation assay to compare diseased patient serum NET degradation capabilities to healthy serum [57]. However, the DHMs greatly differed in structure and distribution in a well from NETs. Moreover, while the image-based method provides insights into how DNA may detach and delaminate from the surface during serum-mediated degradation, it requires a live cell imager. Plate readers are a more ubiquitous tool and more commonly used for NET degradation studies. A total of 20 out of the 24 studies surveyed used plate readers rather than microscopes or other methods (Table 1). Thus, we developed another DNA–histone structure, the surface webs, that is plate-reader-compatible and has a more similar shape, distribution, and protocol to current NET degradation assays (Table 3). The surface webs also have a degradation incubation step of 4 h, which is shorter than the DHMs and within the common range of 1–6 h used for NETs (Table 1). While there is less DNA in the DHMs than in the surface webs, the degradation time for the DHMs is likely higher due to how the DHMs are fabricated, as a singular dense structure, while the surface webs are multiple structures distributed throughout the entire well, more closely resembling NET distribution. Highly dense NETs have been found to cause steric hinderance [62]; therefore, it is possible that the high density of DHMs is limiting degradation.

One of the main limitations of this work is the lack of key NET-associated components beyond DNA and histones. While NETs exhibit high variability based on donor and stimulation method with varying miRNA, proteins, DNA sources, and autoantibodies [31,32,33,34,35], these factors can impact degradation and DNase accessibility. For example, neutrophil elastase is a neutrophil granule commonly found in NETs that partially degrades histone during and after NET formation [4,63]. Neutrophil elastase, together with myeloperoxidase, decondenses the chromatin structure in NETs [21]. There is a tradeoff between accuracy and consistency in the minimal surface web structures, which contain only DNA and histone. On the other hand, we attribute at least a part of the stability of the surface web structure to the lack of elastase [63].

Furthermore, the histones within the surface webs may not have the same post-translational modifications as NETs. NET histones undergo a variety of modifications, including citrullination, methylation, and acetylation [64]. These modifications affect how histone binds to DNA and the overall structure of chromatin. More relaxed, loosened chromatin structures would increase DNase accessibility, leading to more efficient NET clearance. Regardless, the surface webs have been designed to degrade within expected NET degradation timeframes, and, with proper controls, they can provide useful information.

## 5. Conclusions

Interest in NETs as a therapeutic target has been growing, as patients with excessive amounts of NETs have been shown to experience worse disease outcomes. Work has begun to explore treatments that target the DNA in NETs to reduce inflammation in diseases, including respiratory syncytial virus infections and COVID-19 [65,66,67]. Assays for studying NET clearance have been invaluable tools to measure how effectively diseased serum samples can degrade NETs. In the future, these degradation assays could also be used to screen for potential therapeutics to improve NET clearance. However, the throughput of the assay would be limited, as it is difficult to generate NETs at scale and the NETs must be used relatively soon after their formation. In this study, we validate a NET degradation assay mimetic, referred to as surface webs, that can measure serum- and DNase-mediated degradation similarly to NETs while increasing scalability, throughput, ease of use, and lowering costs. The surface webs are well suited for future drug screening and diagnostic research. 

## Figures and Tables

**Table 1:** A non-comprehensive review of NET degradation assay methods.

Citation	Method	Data Reporting Method	Degradation Time	Serum or Plasma %	Negative Control	Positive Control
von Köckritz-Blickwede et al., Blood. 2009 [39]	DNA and myeloperoxidase (MPO) were stained and measured using fluorescence microscopy.	Fluorescence images.	1 h	2%, 5%, and 10% serum (bovine)	Serum-free media	500 mU/mL MNase
Hakkim et al., PNAS. 2010 [40]	DNA was stained and measured using fluorescence spectrometry.	% degradation normalized, healthy neutrophil donor’s serum is 100%.	6 h	10% serum for experiments comparing patient sera, 1, 5, 10, 15, and 20% serum for DNase-mediated degradation experiments		1 U/mL MNase/ DNase I
Leffler et al., J Immunol. 2012 [41]	DNA of degraded NETs that were released into solution was stained and measured with a fluorescence microplate reader.	Some NET degradation experiment data are shown as fluorescence units, some data are shown as the ratio to MNase standard, some data are shown as fold change.	45–60 min	10% serum		50 mU MNase
Leffler et al., Arthritis Res Ther. 2013 [42]	DNA of degraded NETs that were released into solution was stained and measured with a fluorescence microplate reader.	Compared to the mean degradation of pooled healthy sera.	1 h	10% serum		
Leffler et al., Clin Exp Rheumatol. 2014 [28]	DNA of degraded NETs that were released into solution was stained and measured with a fluorescence microplate reader.	Compared to the mean degradation of pooled healthy sera.	1 h	10% serum		
Zhang et al., Clin Exp Immunol. 2014 [43]	DNA was stained and fluorescence was measured with a microplate reader or by manually counting using fluorescence microscopy.	% degradation normalized, negative control is 0% and DNase I positive control is 100%.	2 h	10% plasma	Untreated	0.55 U/mL DNase I
Chauhan et al., Immunol Lett. 2015 [44]	DNA was stained and fluorescence was measured with a microplate reader or by manually counting using fluorescence microscopy.	Relative fluorescence units of DNA released in solution.	1 h	Serum		
Jimmenz-Alcazar et al., J Thromb Haemost. 2015 [45]	DNA was stained and measured with a fluorescence microplate reader or by using fluorescence microscopy.	% DNA remaining normalized, negative control is 100%.	6 h	5% plasma	Buffer	
Leffler et al., Arthritis Res Ther. 2015 [46]	DNA of degraded NETs that were released into solution was stained and measured with a fluorescence microplate reader.	Compared to the mean degradation of pooled healthy sera.	1 h	10% serum		
White et al., J Clin Periodontol. 2016 [47]	DNA of degraded NETs that were released into solution was stained and measured with a fluorescence microplate reader.	% degradation normalized, positive control is 100%.	3 h	10% plasma		1 U/mL MNase
Jimmenz-Alcazar et al., Science. 2017 [15]	DNA was stained and measured with a fluorescence microplate reader or by calculating the area coverage in images.	% NET remaining normalized, negative control is 100%.	6 h	10% serum (murine) or 10% plasma (human)	Buffer	
Leffler et al., J Innate Immun. 2017 [48]	DNA of degraded NETs that were released into solution was stained and measured with a fluorescence microplate reader.	Compared to the mean degradation of pooled healthy sera.	1 h	5–10% serum		
Jeremic et al., Rheumatol Int. 2019 [49]	DNA of degraded NETs that were released into solution was stained and measured with a fluorescence microplate reader.	Concentration of DNA released in solution after degradation.	1 h	10% serum		MNase
Moonen et al., Innate Immun. 2019 [50]	After incubation with patient samples, NET DNA was unbound via incubation with MNase. DNA in the supernatant was stained and measured with a fluorescence microplate reader.	% degradation normalized, positive control is 100%, negative control is 0%.	3 h	10% plasma	Untreated	75 mIU/mL MNase
Zuo et al., Arthritis and Rheumatol. 2020 [29]	DNA was stained and measured with a fluorescence microplate reader.	% degradation	1.5 h	5% serum		10 U/mL MNase
Chen et al., Front Immunol. 2021 [51]	DNA of degraded NETs that were released into solution was stained and measured with a fluorescence microplate reader.	% degradation normalized, negative control is 0%, positive control is 100%.	1 h			
Zuo et al., JCI Insight. 2021 [38]	DNA was stained and measured with a fluorescence microplate reader.	% NET remaining normalized, negative control is 100%.	1.5 h	5% serum	Untreated	10 U/mL MNase
Torres-Ruiz et al., Cells. 2021 [52]	DNA and myeloperoxidase (MPO) were stained and measured with fluorescence microscopy.	Fluorescence images and % degradation normalized, negative control is 0%.	Overnight	10% serum	Untreated	MNase or 1 U/mL DNase
Carmona-Rivera et al., JCI Insight. 2022 [53]	DNA was stained and measured with a fluorescence microplate reader.	Relative fluorescence units of NETs remainingand accompanying fluorescence images.	16 h	5% serum	Untreated	DNase I
Michailidou et al., Clinical Immunology. 2023 [54]	DNA was stained and measured with a fluorescence microplate reader.	Ratio of residual NETs in treated groups to those in untreated groups.	1.5 h	2.5% plasma		MNase
Oliveira et al., J Invest Dermatol. 2023 [55]	DNA was stained and measured with a fluorescence microplate reader.	Relative fluorescence units of NETs remaining and accompanying fluorescence images.	16 h	5% serum	Untreated	
Lipka et al., Plos One. 2023 [56]	DNA was stained and measured with a fluorescence microplate reader.	% degradation normalized, positive control is 100%, percent degraded measured per well.	4, 5, and 21 h	25% serum	Untreated	DNase I
Wasielewski et al., Int J Mol Sci. 2023 [57]	DNA was stained, and the surface area change over time was tracked using live fluorescent imaging.	Some experiment data are shown as the surface area over time, some data are shown as endpoint % remaining calculated from the surface area, some data are shown as fluorescence images.	2, 12, and 24 h	5% serum	Buffer	DNase I
Oto et al., Bone Joint J. 2024 [58]	DNA was stained and measured with a fluorescence microplate reader.	% remaining normalized, negative control is 100%, shown using fluorescence images.	6 h	5% plasma	Buffer	

**Table 2:** A non-comprehensive review of dyes used in NET degradation assays.

Citation	Degradation Calculated Per Well by Comparing Initial to Final?	DNA Stained Pre/Post Degradation Step	Dye
von Köckritz-Blickwede et al., Blood. 2009 [39]		Post	PicoGreen for quantification of NET release, Alexa-488-labeled myeloperoxidase antibodies with DAPI for the degradation assay
Hakkim et al., PNAS. 2010 [40]		Post	PicoGreen
Leffler et al., J Immunol. 2012 [41]		Post	PicoGreen for quantification, propidium iodide or SYTOX Green for visualization
Leffler et al., Arthritis Res Ther. 2013 [42]		Post	PicoGreen
Leffler et al., Clin Exp Rheumatol. 2014 [28]		Post	PicoGreen for quantification, SYTOX Green for visualization
Zhang et al., Clin Exp Immunol. 2014 [43]		Post	SYTOX Green or DAPI
Chauhan et al., Immunol Lett. 2015 [44]		Post	SYTOX Orange
Jimmenz-Alcazar et al., J Thromb Haemost. 2015 [45]		Post	SYTOX Green or anti-DNA antibodies
Leffler et al., Arthritis Res Ther. 2015 [46]			PicoGreen
White et al., J Clin Periodontol. 2016 [47]		Post	SYTOX Green
Jimmenz-Alcazar et al., Science. 2017 [15]		Post	SYTOX Green
Leffler et al., J Innate Immun. 2017 [48]		Post	PicoGreen
Jeremic et al., Rheumatol Int. 2019 [49]		Post	PicoGreen
Moonen et al., Innate Immun. 2019 [50]		Post	SYTOX Green
Zuo et al., Arthritis and Rheumatol. 2020 [29]	Y	Pre	SYTOX Green
Chen et al., Front Immunol. 2021 [51]		Post	PicoGreen
Zuo et al., JCI Insight. 2021 [38]	Y	Pre	SYTOX Green
Torres-Ruiz et al., Cells. 2021 [52]	Y	Pre	Alexa-Fluor-555-labeled MPO antibodies with DAPI
Carmona-Rivera et al., JCI Insight. 2022 [53]		Post	SYTOX Green
Michailidou et al., Clinical Immunology. 2023 [54]	Y	Pre	SYTOX Green
Oliveira et al., J Invest Dermatol. 2023 [55]		Post	SYTOX Green
Lipka et al., Plos One. 2023 [56]	Y	Pre	PicoGreen
Wasielewski et al., Int J Mol Sci. 2023 [57]	Y	Pre	SYTOX Green
Oto et al., Bone Joint J. 2024 [58]		Post	SYTOX Green

**Table 3:** Main differences among NETs, a previously published NET mimetic known as the DHM, and the current proposed surface webs.

	NETs	Previously Published NET Mimetic: DHMs	NET Mimetic: Surface Webs
**Degradation measurement method**	Typically, fluorescence-microplate-reader based. While less frequent, fluorescence microscopy is also used.	Fluorescence microscopy.	Fluorescence microplate reader.
**Time for degradation**	1–21 h	24–48 h	4 h
**Approximate structure shape and distribution**	Multiple structures with longer strands; distributed throughout the bottom of the whole well.	Around 60 μm diameter circular structure; one structure in the center of the well.	Multiple particle structures; evenly distributed throughout the bottom of the whole well.
**DNA type**	Neutrophil nucleus and mitochondrial.	Lambda-phage methylated.	From salmon testes.
**Approximate amount of DNA per well**	180–240 ng from 20–40 k NETotic cells stimulated with 20 nM PMA.	30 ng	250 ng
**Ratio of DNA:Histone (*w*/*w*)**	Approximately 1:1.	1:10	1:1
**Additional components**	Citrullinated histone; neutrophil granule proteins.	Non-citrullinated histone; no additional granule proteins.	Non-citrullinated histone; no additional granule proteins.

Labels in header row and header column are bolded.

## Data Availability

The raw data supporting the conclusions of this article will be made available by the authors upon request.

## Supplementary Materials

The following supporting information can be downloaded at: https://www.mdpi.com/article/10.3390/cells14080615/s1, Table S1. Survey of NET degradation assay methods supplementary; Table S2. *p* values of YOYO-1 stained DNase I wells in Figure 3C; Table S3. *p* values of initial fluoresence readings in Figure 4A; Table S4. *p* values of HC serum #1 conditions in Figure 4B; Table S5. *p* values of HC serum #2 conditions in Figure 4C; Table S6. *p* values of 0.4 U/mL DNase conditions in Figure 4D; Table S7. *p* values of 2 U/mL DNase conditions in Figure 4E; Table S8. *p* values of 5 U/mL DNase conditions in Figure 4F; Table S9. *p* values of 10 U/mL DNase conditions in Figure 4G; Materials List provided for catalog numbers of materials.

## Appendix A. Negative or Above 100% Remaining from Background Subtraction and Calculations

Some figures show a negative % remaining due to the method used for background subtraction and the calculation of % remaining.

All data shown include background subtraction. Background values were obtained from PLL-coated wells with no webs. Wells were washed along with the experimental wells and filled with NB during the degradation assay incubation step. Background values were averaged from readings of the background wells taken during the same step as the experimental wells. Therefore, different values were used for the background in the initial and final readings of the experimental wells. The detailed equation for % remaining with background subtraction is as follows:

(A1) $$\%\;Remaining=\frac{\left(Final\;well\;RFU-average\;final\;background\;RFU\right)}{\left(Initial\;well\;RFU-average\;initial\;background\;RFU\right)}\times 100$$

Occasionally, conditions with high degradation resulted in a final well reading RFU lower than that of the background wells. This led to negative % remaining. Other times, the final RFU was higher than the initial, resulting in a % remaining over 100.
