# Supplementary material for: A Mimetic Assay of Neutrophil Extracellular Trap Degradation Using YOYO-1-Stained DNA-Histone Surface Webs
Source: Cells. 2025 Apr 19;14(8):615. doi: 10.3390/cells14080615 (PMC12025948; doi:10.3390/cells14080615)
Supplement: Supplementary file 1 [file cells-14-00615-s001.zip › Surface web assay_supplementary p values_v3.pdf]

Table S2. P values of YOYO-1 stained DNase I wells in Figure 3C.

Tukey's multiple comparisons test	Below threshold?	Summary	Adjusted P Value
0.4 U/mL DNase vs. 2 U/mL DNase	Yes	****	<0.0001
0.4 U/mL DNase vs. 5 U/mL DNase	Yes	****	<0.0001
0.4 U/mL DNase vs. 10 U/mL DNase	Yes	****	<0.0001
0.4 U/mL DNase vs. NB	Yes	****	<0.0001
2 U/mL DNase vs. 5 U/mL DNase	Yes	****	<0.0001
2 U/mL DNase vs. 10 U/mL DNase	Yes	****	<0.0001
2 U/mL DNase vs. NB	Yes	****	<0.0001
5 U/mL DNase vs. 10 U/mL DNase	Yes	*	0.0123
5 U/mL DNase vs. NB	Yes	****	<0.0001
10 U/mL DNase vs. NB	Yes	****	<0.0001

Table S3. P values of initial fluoresence readings in Figure 4A.

Dunn's multiple comparisons test	Mean rank diff.	Significant?	Summary	Adjusted P Value
0 hr vs. 3 day	-25.7	No	ns	>0.9999
0 hr vs. 7 day	-13.97	No	ns	>0.9999
0 hr vs. 14 day	64.05	Yes	**	0.0036
0 hr vs. 30 day	133.7	Yes	****	<0.0001
3 day vs. 7 day	11.73	No	ns	>0.9999
3 day vs. 14 day	89.75	Yes	****	<0.0001
3 day vs. 30 day	159.4	Yes	****	<0.0001
7 day vs. 14 day	78.03	Yes	***	0.0001
7 day vs. 30 day	147.7	Yes	****	<0.0001
14 day vs. 30 day	69.69	Yes	**	0.001

Table S4. P values of HC serum #1 conditions in Figure 4B.

Dunn's multiple comparisons test	Mean rank diff.	Significant?	Summary	Adjusted P Value
0 day vs. 3 day	3.857	No	ns	>0.9999
0 day vs. 7 day	0.4286	No	ns	>0.9999
0 day vs. 14 day	-6.714	No	ns	>0.9999
0 day vs. 30 day	-8.286	No	ns	>0.9999
3 day vs. 7 day	-3.429	No	ns	>0.9999
3 day vs. 14 day	-10.57	No	ns	0.536
3 day vs. 30 day	-12.14	No	ns	0.2662
7 day vs. 14 day	-7.143	No	ns	>0.9999
7 day vs. 30 day	-8.714	No	ns	>0.9999
14 day vs. 30 day	-1.571	No	ns	>0.9999

Table S5. P values of HC serum #2 conditions in Figure 4C.

Dunn's multiple comparisons test	Mean rank diff.	Significant?	Summary	Adjusted P Value
0 day vs. 3 day	12	No	ns	0.2846
0 day vs. 7 day	1.857	No	ns	>0.9999
0 day vs. 14 day	2	No	ns	>0.9999
0 day vs. 30 day	3.429	No	ns	>0.9999
3 day vs. 7 day	-10.14	No	ns	0.6405
3 day vs. 14 day	-10	No	ns	0.6789
3 day vs. 30 day	-8.571	No	ns	>0.9999
7 day vs. 14 day	0.1429	No	ns	>0.9999
7 day vs. 30 day	1.571	No	ns	>0.9999
14 day vs. 30 day	1.429	No	ns	>0.9999

Table S6. P values of 0.4 U/mL DNase conditions in Figure 4D.

Dunn's multiple comparisons test	Mean rank diff.	Significant?	Summary	Adjusted P Value
0 day vs. 3 day	11.86	No	ns	0.304
0 day vs. 7 day	-1.143	No	ns	>0.9999
0 day vs. 14 day	-9.571	No	ns	0.8055
0 day vs. 30 day	-10.43	No	ns	0.5691
3 day vs. 7 day	-13	No	ns	0.1762
3 day vs. 14 day	-21.43	Yes	***	0.0009
3 day vs. 30 day	-22.29	Yes	***	0.0005
7 day vs. 14 day	-8.429	No	ns	>0.9999
7 day vs. 30 day	-9.286	No	ns	0.9001
14 day vs. 30 day	-0.8571	No	ns	>0.9999

Table S7. P values of 2 U/mL DNase conditions in Figure 4E.

Dunn's multiple comparisons test	Mean rank diff.	Significant?	Summary	Adjusted P Value
0 day vs. 3 day	12.43	No	ns	0.2326
0 day vs. 7 day	3.429	No	ns	>0.9999
0 day vs. 14 day	-9.714	No	ns	0.7613
0 day vs. 30 day	-14	No	ns	0.1059
3 day vs. 7 day	-9	No	ns	>0.9999
3 day vs. 14 day	-22.14	Yes	***	0.0005
3 day vs. 30 day	-26.43	Yes	****	<0.0001
7 day vs. 14 day	-13.14	No	ns	0.1642
7 day vs. 30 day	-17.43	Yes	*	0.0146
14 day vs. 30 day	-4.286	No	ns	>0.9999

Table S8. P values of 5 U/mL DNase conditions in Figure 4F.

Dunn's multiple comparisons test	Mean rank diff.	Significant?	Summary	Adjusted P Value
0 day vs. 3 day	12.71	No	ns	0.2027
0 day vs. 7 day	4	No	ns	>0.9999
0 day vs. 14 day	-9.571	No	ns	0.8055
0 day vs. 30 day	-13.57	No	ns	0.1322
3 day vs. 7 day	-8.714	No	ns	>0.9999
3 day vs. 14 day	-22.29	Yes	***	0.0005
3 day vs. 30 day	-26.29	Yes	****	<0.0001
7 day vs. 14 day	-13.57	No	ns	0.1322
7 day vs. 30 day	-17.57	Yes	*	0.0134
14 day vs. 30 day	-4	No	ns	>0.9999

Table S9. P values of 10 U/mL DNase conditions in Figure 4G.

Dunn's multiple comparisons test	Mean rank diff.	Significant?	Summary	Adjusted P Value
0 day vs. 3 day	15	No	ns	0.0617
0 day vs. 7 day	11.43	No	ns	0.3693
0 day vs. 14 day	4.286	No	ns	>0.9999
0 day vs. 30 day	-7.857	No	ns	>0.9999
3 day vs. 7 day	-3.571	No	ns	>0.9999
3 day vs. 14 day	-10.71	No	ns	0.5045
3 day vs. 30 day	-22.86	Yes	***	0.0003
7 day vs. 14 day	-7.143	No	ns	>0.9999
7 day vs. 30 day	-19.29	Yes	**	0.0043
14 day vs. 30 day	-12.14	No	ns	0.2662
